# DDR-coin: An Efficient Probabilistic Distributed Trigger Counting Algorithm

**DOI:** 10.3390/s20226446

**Published:** 2020-11-11

**Authors:** Seokhyun Kim, Yongsu Park

**Affiliations:** 1 Coupang Corp., Tower 730, 570 Songpa-daero, Songpa-gu, Seoul 05510, Korea; seokhyun.kim.78@gmail.com; 2 Department of Computer Science, Hanyang University, Seoul 04763, Korea

**Keywords:** distributed trigger counting, distributed algorithm, probabilistic algorithm, distributed systems

## Abstract

A distributed trigger counting (DTC) problem is to detect *w* triggers in the distributed system consisting of *n* nodes. DTC algorithms can be used for monitoring systems using sensors to detect a significant global change. When designing an efficient DTC algorithm, the following goals should be considered; minimizing the whole number of exchanged messages used for counting triggers and even distribution of communication loads among nodes. In this paper, we present an efficient DTC algorithm, DDR-coin (Deterministic Detection of Randomly generated coins). The message complexity—the total number of exchanged messages—of DDR-coin is $O(n\log_{n}\left(w/n\right))$ in average. MaxRcvLoad—the maximum number of received messages to detect *w* triggers in each node—is $O(\log_{n}\left(w/n\right))$ on average. DDR-coin is not an exact algorithm; even though *w* triggers are received by the *n* nodes, it can fail to raise an alarm with a negligible probability. However, DDR-coin is more efficient than exact DTC algorithms on average and the gap between those is increased for larger *n*. We implemented the prototype of the proposed scheme using NetLogo 6.1.1. We confirmed that experimental results are close to our mathematical analysis. Compared with the previous schemes—TreeFill, CoinRand, and RingRand— DDR-coin shows smaller message complexity and MaxRcvLoad.

## 1. Introduction

Consider a distributed system with sensors, e.g., the wireless sensor network (WSN). For many cases, monitoring is one of the most important issues and the system would like to detect a significant global state change. For example, we consider traffic surveillance where a large number of sensors are distributed in a targeted area. When the predefined number of cars have passed the targeted area, the system raises an alarm. Another example is that a large number of illegal login attempts on diverse nodes should be alarmed.

A distributed trigger counting (DTC) problem can play an important role in this kind of monitoring applications. DTC problem is formally defined as follows. Suppose a distributed system where *n* nodes communicate with each other. Assume that from external sources, *w* triggers arrive at the *n* nodes, and that no statistical information about the triggers is given to the system in advance. We consider the case where the number of triggers is much greater than the number of nodes, i.e., w≫n (If w≤n, the number of triggers can be easily aggregated using a spanning tree of nodes [1,2,3]). The distributed trigger counting (DTC) problem is to raise an alarm when the total number of detected triggers by the *n* nodes reaches to *w*.

In a distributed system, various state changes or data from sensors can be used to initiate a trigger. Thus, if we define a global threshold for a certain property on a distributed system as the number of total generated triggers in the system, DTC algorithms can be useful for detecting the time when the global threshold is reached, e.g., in the above traffic surveillance example, we can use a DTC algorithm for counting the number of cars passing that area: When *w* cars have passed the targeted area, the system raises an alarm. Moreover, DTC algorithms play an important role in many distributed applications including taking global snapshots and monitoring significant events, e.g., in conventional global snapshot algorithms, the total number of exchanged messages to take a global snapshot is $O(n^{2})$ [4,5,6]. With DTC algorithms, the number of exchanged messages can be reduced significantly (for more detailed relationship between DTC algorithms and global snapshot algorithms, please refer to the work in [3]). DTC algorithms can also be useful for monitoring specific events [7,8,9,10,11], which is one of the core functionalities in distributed systems including computational grids, cluster computing and wireless sensor networks [12,13,14,15,16].

An exact DTC algorithm should always raise an alarm when *w* triggers have arrived at *n* nodes whereas a probabilistic one fails sometimes (to be practical, the failure probability should be very low). Garg et al. showed that the lower bounds on message complexity (i.e., total number of exchanged messages) of an exact DTC algorithm are Ω(nlog(w/n)) [3]. Moreover, they showed that the lower bounds on MaxRcvLoad (i.e., the maximum number of received messages in each node) is Ω(log(w/n)) [3]. Garg et al. suggested the tree-based DTC algorithm and the centralized one, both of which are exact algorithms [3]. Their centralized algorithm has optimal message complexity but MaxRcvLoad is high (and not analyzed). Chakaravarthy et al. suggested the sub-optimal DTC algorithm for both total message complexity and MaxRcvLoad, where their algorithm uses a tree-like network topology [2]. Kim et al. suggested an optimal DTC algorithm [17] in terms of message complexity and MaxRcvLoad, where its algorithm is more complex than [2]. Emek et al. improved lower bounds on DTC algorithms and proposed the probabilistic DTC algorithm, where its message complexity is low but MaxRcvLoad is not bounded [18]. Chang et al. suggested the DTC algorithm that can work with any network topology [19].

In this paper, we present an efficient probabilistic DTC algorithm, DDR-coin (Deterministic Detection of Randomly generated coins). DDR-coin has a (one-sided) failure probability, where the failure probability is defined as the probability of not raising an alarm even if the number of triggers reaches *w*. Table 1 summarizes the comparison results between previous work and DDR-coin. We use the following performance metrics to evaluate our algorithm, which are also used in previous work [1,2,3,17].

- **Message complexity**: the total number of exchanged messages among the nodes. For efficiency, this should be low.
- **MaxMsgLoad**: the maximum number of exchanged (i.e., sent and received) messages in each node. For even distribution of load, this should be low.
- **MaxRcvLoad**: the maximum number of received messages in each node. For even distribution of load, this should be low.

As seen in Table 1, the average message complexity of DDR-coin is $O(n\log_{n}\left(w/n\right))$, which is lower than the optimal message complexity (O(nlog(w/n)) [17]) of exact DTC algorithms. The MaxRcvLoad of DDR-coin is $O(\log_{n}\left(w/n\right))$ on average, which is lower than those of other schemes. (For MaxMsgLoad, just as in many of previous schemes, we were unable to get the bounds of DDR-coin since it is too complex.) The failure probability of DDR-coin is negligible, which will be seen in Section 3.1.3. We implemented the prototype of the proposed scheme using NetLogo 6.1.1. We confirmed that experimental results are close to our mathematical analysis. Compared with the previous schemes—TreeFill, CoinRand, and RingRand— DDR-coin shows smaller message complexity and MaxRcvLoad.

This paper is organized as follows. The DDR-coin algorithm is explained in Section 2. We analyze the failure probability, message complexity, and MaxRcvLoad of DDR-coin in Section 3. We show experimental results in Section 4. The related works on DTC algorithms are summarized in Section 5. We conclude this paper in Section 6.

## 2. DDR-coin Algorithm

After we describe the system model and our objectives in Section 2.1, an overview of DDR-coin is given in Section 2.2. Section 2.3 explains the tree-like structure used by DDR-coin and Section 2.4 deals with detailed explanation of the DDR-coin algorithm. Table 2 summarizes explanation on notation used in this paper.

### 2.1. System Model and Objectives

We assume that the number of nodes in the system is *n*. To simplify the problem, assume that the nodes are fully connected, there are no message drops, there are no external attackers, and the nodes do not fail. Events are being triggered with arbitrary distribution on these nodes in the system. We want to detect and raise an alarm when *w* or more triggers occur in the system. To this end, *n* nodes should send and receive messages, and we want to minimize this (i.e., minimizing message complexity). We also want communication overheads to be evenly distributed among nodes (i.e., minimizing MaxRcvLoad). We assume that events continue to be triggered while the protocol is running.

We only consider the case where the number of triggers is much greater than the number of nodes, i.e., w≫n (for w≤n, the works in [1,2,3] solve the problem with O(n) messages using spanning trees).

Our objectives are as follows.

- When *w* or more than *w* triggers occur, the system has a very high probability of raising an alarm. (In other words, the failure probability is negligible.)
- When the system raises an alarm, the probability that the number of triggers is less than *w* is 0 (i.e., no false positives).
- The average message complexity is $O(n\log_{n}\left(w/n\right))$.
- The average MaxRcvLoad is $O(\log_{n}\left(w/n\right))$.

### 2.2. Overall of DDR-coin

**Figure 1:**
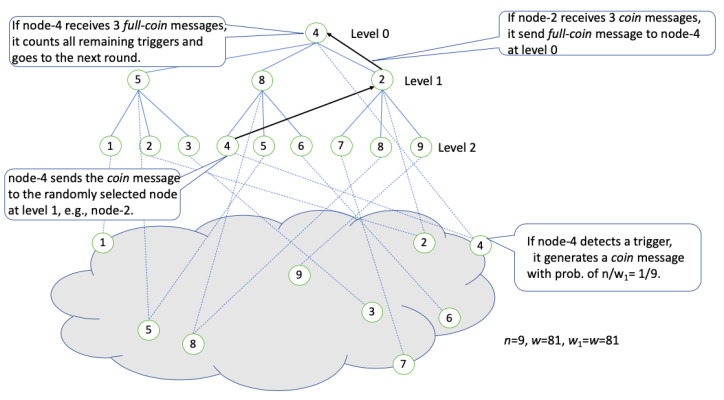
An example of DDR-coin in round 1 when *n* = 9 and *w* = 81.

The system works in the following way. *n* nodes have hierarchy to form a complete tree-like structure, e.g., the lower part of Figure 1 shows the nodes on the network when *n* = 9, and the upper part corresponds to the tree-like structure of these nodes. All the nodes correspond to leaf vertices at the level-*h* of the tree (*h*: the height of the tree-like structure), and some nodes correspond to internal vertices in addition to the leaves (i.e., have dual roles). The tree-like structure will be explained in detail in Section 2.3.

DDR-coin operates in multi-round. For Round 1, $w_{1}$, the number of remaining triggers to be detected is set to *w*. The goal of Round 1 is to detect the state where nodes have been received slightly less than $w_{1}$ triggers.

To do so, when an event is triggered on the node associated with the leaf with level-*h*, with a specific probability, a message is sent to a node associated with the internal vertex corresponding to *h*-1 level. Then, the node corresponding to the level *h*-1 counts the number of received messages, and when it exceeds a certain threshold, this node sends a message to a node corresponding to the level *h*-2 to inform reaching the threshold. When we repeat the work in this way, the node corresponding to the root (level-0) finally receives messages from nodes at level-1. Then, the root starts the aggregating work that counts the number of triggers that have occurred in all nodes (which we call the end-of-round procedure).

In the end-of-round procedure, the root propagates the aggregating message to the leaves and then each leaf sends the message that contains the number triggers (i.e., events triggered) in the leaf to the root. At the end of this process, the root node knows that $\hat{w_{1}}\left(\leq w_{1}\right)$ triggers have occurred in Round 1. Then, Round 1 is finished and Round 2 starts. Round 2 works in the same way, but the threshold/parameters are adjusted to detect slightly less than $w_{2}\left(=w_{1}-\hat{w_{1}}\right)$ triggers.

If we repeat this work, the number of remaining triggers will gradually decrease and will be less than or equal to *n*. Then, it goes to the final round: using the procedure of Section 2.4.4, we count the number of triggers that have occurred exactly *w* and raise an alarm.

For better understanding, a detailed example for Rounds 1 and 2 is given in Appendix A.

### 2.3. Tree-Like Structure

In this section, we describe the tree-like structure used by DDR-coin. This structure is the complete *k*-ary tree, where vertices are associated with nodes in the network. An example of the tree-like structure in DDR-coin, when k=3 and n=9, is shown in the upper side of Figure 1.

We define level-*l* as follows; the root vertex is in level-0 and the vertices at level-(l+1) are children of vertices at level-*l*. Note that all the *n* nodes are related with *n* leaf vertices in level-*h*, where *h* is the height of this tree-like structure, i.e., the maximal level, e.g., in Figure 1, h=2. Internal vertices are from level-0 through level-(*h*-1). We assume $n=k^{h}$ for ease of algorithm explanation and analysis. Our algorithm can be easily extended to general cases (which may be hard to analyze mathematically).

Each node in the network (e.g., at the bottom of Figure 1) is associated with each leaf vertex in this tree-like structure. For example, in Figure 1 below, node-3 in the network is associated with leaf vertex-3 of the tree-like structure. Some nodes have dual roles: a node is associated with one leaf and one internal vertex, e.g., in Figure 1, node-4 in the network is associated with leaf vertex-4 and root vertex-4. From now on, “the node *u* in the tree-like structure” denotes the node *u* in the network where the node *u* is associated with the vertex *u* of the tree-like structure.

Actually, we use this tree-like structure to associate the level of a tree with a node but the message is not necessarily transmitted along the edge of the tree, e.g., as will be explained in detail in Section 2.4, a node at level *l* sends a message to any node at level l−1/l+1 as well as parent/children.

At the beginning the DDR-coin protocol, the nodes for internal vertices are chosen among the *n* nodes, e.g., in Figure 1, node-4, -5, -8, and -2 are chosen to be internal vertices. Even though we can select any nodes to be the internal vertices, one simple approach may be selecting first (n−1)/(k−1) nodes for the internal vertices.

### 2.4. DDR-coin Algorithm

DDR-coin works based on rounds. Overall operations in DDR-coin are as follows. Steps 1–3 are for each round and Step 4 is for the final round.

1. **(Coin generation routine)** Recall that all *n* nodes are associated at leaf-level (level-*h*). When a node detects a trigger, it generates a *coin* message with the probability of $n/w_{i}$, where $w_{i}$ is the number of not yet received triggers at the beginning of round *i*. (Initially $w_{1}=w.$) This *coin* message is sent to the randomly-selected node at level-(*h*-1).
2. **(Coin propagation routine)** The *coin* messages are propagated from leaves in the tree-like structure to the root. Eventually, the node for the root vertex detects that *n* coins have been generated at the leaf-level.
3. **(End-of-round procedure)***n* nodes count the number of generated triggers up to now (using the spanning tree). If the number of not yet detected triggers is greater than *n*, a new round starts by going back to the coin generation routine again (Step 1). Otherwise, it goes to the final round procedure (Step 4).
4. **(Final round procedure)** It counts the remaining triggers (the number of which does not exceed *n*). Then, it raises an alarm.

#### 2.4.1. Coin Generation Routine

Let $w_{i}$ be the number of the triggers that are not yet detected at the beginning of *i*-th round. When *i*-th round begins, $w_{i}$ is calculated as follows; $w_{1}=w$ and $w_{i}=w_{i-1}-\hat{w_{i-1}}\left(i\geq 2\right)$, where $\hat{w_{i-1}}$ is the number of counted (i.e., detected) triggers in (i−1)th round.

Let $n_{j}\left(1\leq j\leq n\right)$ be a node in the system and $n_{j}.trg$ be the number of received triggers in $n_{j}$. Initially, $n_{j}.trg\left(1\leq j\leq n\right)$ is set to be zero.

**Figure 2:**
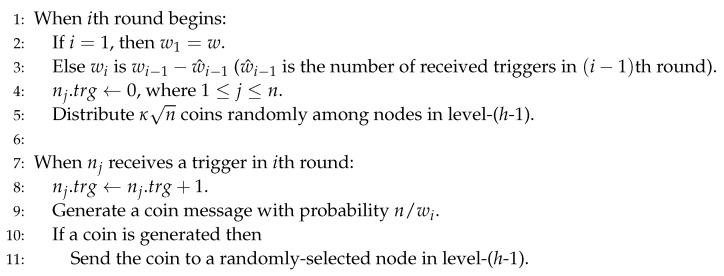
Coin generation routine for node $n_{j}\left(1\leq j\leq n\right)$ in *i*th round.

When $n_{j}$ receives a trigger, it increases $n_{j}.trg$ by one and generates a coin message with the probability of $n/w_{i}$. The coin is sent to a randomly-selected node in level-(*h*-1) of the tree-like structure (note that vertices for all the *n* nodes are in level-*h* so coins are sent from level-*h* to *h*-1), e.g., in Figure 1, if node-4 detects a trigger, node-4.trg= node-4.trg+1, it generates a coin message with the probability of $n/w_{1}=1/9$ and then it sends this coin to a randomly-selected node, e.g., node-2 at level-1. Figure 2 shows the algorithm for the coin generation routine.

#### 2.4.2. Coin Propagation Routine

**Figure 3:**
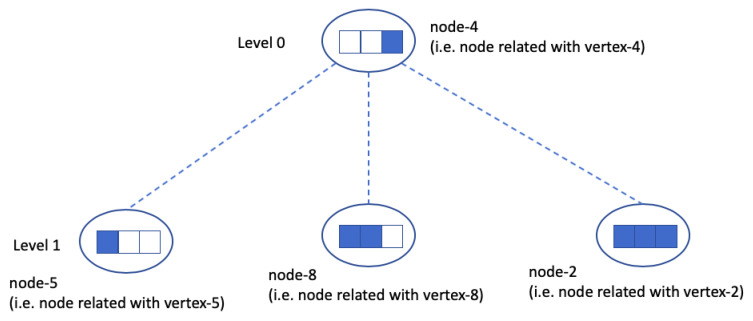
The internal vertices of Figure 1. Circles represent nodes and boxes in circles represent arrays cns[]. The filled box means true while the empty box means false. Currently, 6 coins have arrived at level-(*h*-1) (from level-*h*): 1 for node-5, 2 for node-8, and 3 for node-2. In the root node, node-4, node-4.cns[3]=1. This means that the third subtree of node-4 is fully filled with coins.

The goal of the coin propagation routine is that the node for the root vertex detects that *n* coins have been generated at the leaves. Let $d_{u}$ be a node for internal vertex from level-0 to *h*-1 (1≤u≤(n−1)/(k−1)). e.g., Figure 3 shows internal vertices (node-4, node-5, node-8, and node-2) of tree-like structure of Figure 1.

Each $d_{u}$ has a Boolean array of length *k*, $d_{u}.cns\left[1..k\right]$. This array is initialized with false values at the beginning of a round. This array has two meanings. First, recall that in Section 2.4.1, coins are sent to the node $d_{u}$ at level-(*h*-1). In level-(*h*-1), if $d_{u}$ receives a *v*-th coin, $d_{u}.cns\left[v\right]$(1≤v≤k) becomes true. In this way, the array means $d_{u}$ has received *v* coins from the node at leaf-level (level-*h*), e.g., in Figure 3, currently 6 coins have arrived to the level-1: node-5 has one coin, node-8 has two coins, and node-2 has three coins.

Second, for node $d_{u}$ from level-0 to *h*-2, if $d_{u}.cns\left[v\right]$(1≤v≤k) is true, it means that all the nodes in the *v*-th subtree of $d_{u}$ are fully filled with coins (i.e., all entries in the arrays are true), e.g., Figure 3 shows that node-4 has set node-4.cns[3] as true, because the third subtree of node-4 (i.e., node-2) has fully filled with k=3 coins.

When a coin arrives at $d_{u}$ at level *h*-1, there are 3 cases:

1. If a coin arrives and $d_{u}.cns\left[1\ldots k\right]$ is not full (i.e., some entries are false), one entry with false is changed to true.
2. Suppose that $d_{u}$ has received k−1 coins. When a new coin arrives at $d_{u}$, now $d_{u}.cns\left[1\ldots k\right]$ becomes full (i.e., all entries are true). $d_{u}$ sends a *full-coin* to its parent. Then, the parent node of $d_{u}$ sets the *j*-th entry of cns[1…k] as true where $d_{u}$ is *j*-th child, e.g., in Figure 3, when a new coin is sent to node-8, a *full-coin* is sent to node-4 and node-4.cns[2] is set true. If the parent’s array now also becomes full, the *full-coin* is sent to the grandparent. This work can be repeated until the level-0.
3. If a coin arrives at $d_{u}$ where $d_{u}.cns\left[1\ldots k\right]$ are already all true, $d_{u}$ sends an *overflow-coin* to the randomly chosen node $d_{upper}$ at the upper level. After receiving this, $d_{upper}$ finds a subtree *j* where $d_{upper}.cns\left[j\right]$ is false. This means the corresponding subtree is not fully filled with coins. (If not found, i.e., full, $d_{upper}$ sends this coin to randomly selected node of $d_{upper}$’s upper level.) $d_{upper}$ sends *overflow-coin* down to the root of *j*-th subtree. In this way, the *overflow-coin* is going down again and it is eventually sent down to the node $d_{v}$ at level-(h-1) and $d_{v}$ puts this coin in the array $d_{v}.cns\left[1\ldots k\right]$: false value in the entry is changed to true, e.g., when a new coin is sent to node-2 of Figure 3, because node-2.cns[1…k] is already full, the new *overflow-coin* is sent to a randomly selected node in its upper level, e.g., in Figure 3, there is only node-4 in the upper level of node-2, and the *overflow-coin* is sent to node-4. In Figure 3, node-4 knows that node-2 is full with 3 coins as node-4.cns[3] is true, and node-5 and node-8 have rooms for another coins. node-4 forwards *overflow-coin* to node-5. After node-5 receives the forwarded *overflow-coin*, node-5.cns[2]=true.

**Figure 4:**
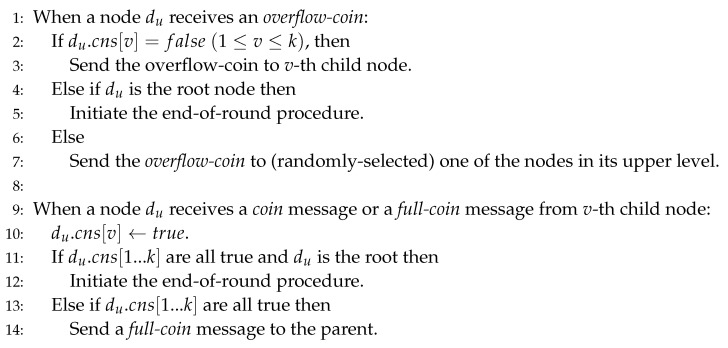
The algorithm of coin propagation routine.

This process continues until all the nodes in the level-(*h*-1) vertices are fully filled with coins, where the number of those coins is *n*. If fully filled, in the node for the root, $d_{root}.cns\left[1\ldots k\right]$ are all true and the root initiates the end-of-round procedure. Figure 4 shows the algorithm of the coin propagation routine.

To increase the probability of going to the end-of-round procedure, in the beginning of each round, after all arrays $d_{u}.cns\left[\right]$ are initialized with false, $\kappa \sqrt{n}$ coins are randomly predistributed among the nodes at level-(h-1) in advance, i.e., $\kappa \sqrt{n}$ entries in arrays are true (which is described in Line 5 of Figure 2). $\kappa (\ll \sqrt{n})$ is a security parameter to adjust the failure probability. We will analyze the relation of κ and the failure probability in Section 3.1.

#### 2.4.3. End-of-Round Procedure

In the end-of-round procedure, the root node sends aggregation-request messages to its children nodes. These messages are recursively sent to the leaf nodes in level-*h*.

Recall that all the *n* nodes are in level-*h*. Each node $n_{j}\left(1\leq j\leq n\right)$ sends the count-message containing the number of received triggers (=$n_{j}.trg$) to its parent node. The internal nodes of DDR-coin aggregate the number of received triggers sent from its children nodes and send the sum to its parent node. Finally, the total number of received triggers at round *i*, $\hat{w_{i}}$, can be calculated at the root node of DDR-coin.

**Figure 5:**
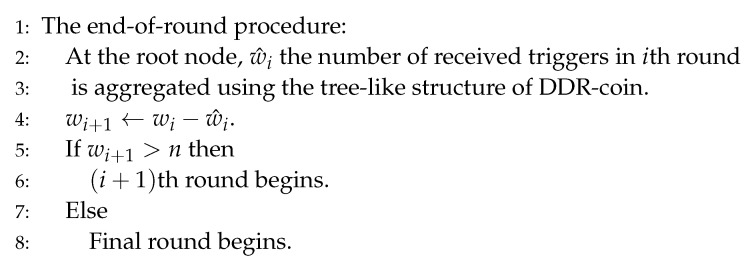
The end-of-round procedure.

Let the number of received triggers by *n* nodes in *i*th round be ${\hat{w}}_{i}$. Then, in the root node, $w_{i+1}$ is calculated as follows; $w_{i+1}=w_{i}-{\hat{w}}_{i}$. If $w_{i+1}>n$, the probability to generate a coin is changed to $n/w_{i+1}$ and (i+1)th round begins. If $w_{i+1}\leq n$, the final round begins. Figure 5 shows the algorithm for the end-of-round procedure.

#### 2.4.4. Final Round Routine

Let $w_{f}\leq n$ be the number of not yet detected triggers in the beginning of the final round. In the beginning of the final round, $n-w_{f}$ coins are distributed among the nodes of level-(h-1) in advance. In each node in level-*h*, the coin generating probability is set to one, i.e., each node generates a coin whenever it receives a trigger.

When $w_{f}$ coins are generated in the nodes in level-*h*, the number of coins in the nodes at level-(*h*-1) is $\left(n-w_{f}\right)+w_{f}=n$ and the root of DDR-coin detects this and raises an alarm.

## 3. Analysis

In this section, we show that (1) when *w* or more than *w* triggers occur, the system detects this with a very high probability and raises an alarm (i.e., the failure probability is negligible). (2) When the system raises an alarm, the probability that the number of triggers is less than *w* is zero. (3) The average message complexity is $O(n\log_{n}\left(w/n\right))$. (4) The average MaxRcvLoad is $O(\log_{n}\left(w/n\right))$. As discussed in Section 4.4 in detail, we conduct analysis under the assumption that $\kappa (\ll \sqrt{n})$ is a small constant positive integer (e.g., 4∼6).

### 3.1. Failure Probability

The success probability is defined as the probability that the system raises an alarm when *w* or more triggers have occurred. The failure probability is the probability that it fails to raise an alarm for this case, which is equal to 1—(success probability).

DDR-coin operates in multi-round, and raises an alarm when, in the last round, the number of triggers that the root node has counted is not less than *w*. Therefore, failure means that it stops before the last round or a problem occurs in the last round. The success probability can be derived by multiplying the probability of successful execution of each round. If the success probability is obtained, the failure probability can be easily calculated.

We first calculate the failure probability of each round and then we calculate the average number of rounds. From this, we will obtain the success/failure probability of DDR-coin.

#### 3.1.1. Failure Probability for each Round

In this subsection, we calculate the failure probability for each round. Each round works as follows. First, $\kappa \sqrt{n}$ coins are randomly sent to the nodes at level-(*h*-1) of the tree-like structure in advance. Then, for each trigger, a coin is generated with a specific probability (and then is sent from level-*h* to level-(*h*-1)). When *n* coins are in the nodes at level-(*h*-1), the root node detects this (by checking that the array of the root node is full) and goes to the end-of-round procedure. Then, it goes to the next round.

Because the end-of-round routine eventually finishes for every case, the failure occurs in each round when the leaf nodes at level-*h* have generated less than $n-\kappa \sqrt{n}$ coin messages, which implies that the root node’s array is not full and the root is waiting forever. Therefore, the failure probability for each round is defined as follows; when the number of observed triggers in the *n* nodes is $w_{i}$, the probability of less than $n-\kappa \sqrt{n}$ *coin* messages are generated.

We show that the failure probability for each round is negligible. Let $w_{i}$ triggers has observed during round *i* (1≤i). The random variable *X* denotes the number of generated coins in *i*-th round. Theorem 1 shows that $Pr(X<n-\kappa \sqrt{n})$ is negligible with the security parameter κ.

**Theorem** **1.**  *When $w_{i}$ triggers have been observed during round i, the probability of generating less than $n-\kappa \sqrt{n}$* coin *messages is negligible: $Pr\left(X<n-\kappa \sqrt{n}\right)<\exp^{-\frac{\kappa^{2}}{2}}$.*

**Proof.**  $e_{1},\ldots ,e_{w_{i}}$ denote the triggers. Recall that for each trigger, a coin message is generated with the probability of $\frac{n}{w_{i}}$ (independent event). Let $X_{k}\left(1\leq k\leq w_{i}\right)$ be the binary random variable describing for generating a coin message, which is a Bernoulli trial. (1 means successfully generated, 0 means not generated.) $X=\sum_{k=1}^{w_{i}}X_{k}$. E(X)=n. By Chernoff–Hoeffding bounds [20], $Pr\left(X<n-\kappa \sqrt{n}\right)=Pr\left(X<\left(1-\kappa /\sqrt{n}\right)n\right)<e^{-n(\kappa^{2}/n)/2}=e^{-\kappa^{2}/2}.$ □

Theorem 1 shows that DDR-coin eventually finishes the round with the negligible failure probability, e.g., if κ=5, this probability is $3.726*10^{-6}.$

#### 3.1.2. The Average Number of Rounds

We show that the average number of rounds in DDR-coin is $O(\log_{n}\left(w/n\right))$ by Theorem 2.

**Theorem** **2.**  *If i-th round finishes with $n-\kappa \sqrt{n}$* coin *messages generated by triggers at the leaf-level (level-h), the average number of triggers is $\left(1-\kappa /\sqrt{n}\right)w_{i}$.*

**Proof.**  $e_{1},\ldots ,e_{w_{i}}$ denote the triggers. Recall that for each trigger, the coin message is generated with the probability of $p=\frac{n}{w_{i}}$ (independent event). Suppose that $n-\kappa \sqrt{n}$ coin messages have been generated. Let *Y* be the number of triggers. *Y* has Pascal distribution (also known as negative binomial distribution) [21]. The expectation, E[Y], is $\left(n-\kappa \sqrt{n}\right)/p=\left(1-\kappa /\sqrt{n}\right)w_{i}.$ □

By Theorem 2, after *i*th round of DDR-coin, the average number of remaining triggers to be counted is ${\left(\kappa /\sqrt{n}\right)}^{i}w$. At the begining of the final round *f*, $w_{f}={\left(\kappa /\sqrt{n}\right)}^{f-1}w$ in average. In the final round, in the worst case, $w_{f}=n$. Therefore, the average number of rounds in DDR-coin is $O(\log_{n}\left(w/n\right))$.

#### 3.1.3. Failure Probability and Success Probability

By Theorems 1 and 2, we get the failure probability of DDR-coin as follows: if the number of observed triggers is *w*, DDR-coin detects this with negligible failure probability: $1-{\left(1-e^{-\kappa^{2}/2}\right)}^{O(\log_{n}\left(w/n\right))}$, e.g., if n=200,w=10,000, and κ=5, the number of rounds in DDR-coin is 5 and the failure probability is $1.863*10^{-5}$. The success probability is 1—the failure probability.

### 3.2. False Positive Probability

In this paper, the false positive probability means the probability that the number of triggers is less than *w* when the system raises an alarm. In DDR-coin, the false positive probability is 0 as DDR-coin counts the number of triggers that have occurred in the final round and generates an alarm only when the total number is not less than *w*.

### 3.3. Message Complexity

In the *i*th round of DDR-coin, the total number of messages exchanged among nodes is the summation of the following.

(i) The number of coins (=$\kappa \sqrt{n}$), which are predistributed in the nodes at level-(h-1) in advance.
(ii) The average number of generated coins from the *n* leaf nodes at level-*h*.
(iii) The average number of *overflow-coin* messages.
(iv) The number of *full-coin* messages.
(v) The number of trigger-aggregation messages at the end-of-round procedure.

In the above numbers, (i), (ii), (iv), and (v) are all O(n), i.e., (i): $\kappa \sqrt{n}$, (ii): $n-\kappa \sqrt{n}$ in average, (iv): each internal node receives one full-coin message and the number of internal nodes is (n−1)/(k−1), (v): the number of aggregation-request messages is the number of edges in tree-like structure: (n−1)/(k−1)+n−1 and the number of count-messages is the same: (n−1)/(k−1)+n−1.

By Appendix B, (iii) the average number of *overflow-coin* messages is O(n). Therefore, the number of messages exchanged among nodes in *i*th round of DDR-coin is O(n) on average.

We already showed that the number of rounds in DDR-coin is $O(\log_{n}\left(w/n\right))$ in Section 3.1.2. Therefore, the overall message complexity of DDR-coin is $O(n\log_{n}\left(w/n\right))$ on average.

### 3.4. MaxRcvLoad

In this subsection, we show that MaxRcvLoad of DDR-coin is $O(\log_{n}\left(w/n\right))$ with the exponentially high probability when k=2. In the *i*th round of DDR-coin, the maximal number of messages in a node is the summation of the following.

(i) The number of predistributed coins.
(ii) The number of generated coins from the *n* leaf nodes at level-*h*.
(iii) The number of *overflow-coin* messages.
(iv) The number of *full-coin* messages.
(v) The number of trigger-aggregation messages at the end-of-round procedure.

In the above numbers, (i)+(ii): *n* coins are independently arrive at *n* node in average and the probability of receiving more than 2 coins in a node is Pr(X≥(1/n+1/n)n)≤exp(−2/n) by Chernoff–Hoeffding bounds [20]. (iv): each internal node receives one full-coin message so 1 is maximum for each node. (v): the maximum number of aggregation-request messages sent/received in each node is *k*. That of the count-messages is the same: *k*.

By Appendix B, (iii) each node at level-*j* forwards a overflow coin to upper level (*j*-1) with the probability of less than 1/2 where the number of nodes at the upper level (*j*-1) is 1/*k* times smaller than that of level-*j*. This implies that among all nodes, the root node receives the maximum number of overflow coins: $n*{\left(\frac{1}{2}\right)}^{h-1}$, where $h=log_{k}\left(n\right)$, i.e., $n{\left(\frac{1}{2}\right)}^{log_{k}n-1}$.

By summing (i)–(v) and then multiplying the average number of rounds, we get MaxRcvLoad as follows: $\left(2+n{\left(\frac{1}{2}\right)}^{log_{k}n-1}+1+2k\right)\left(O\left(\log_{n}\left(w/n\right)\right)\right)=O\left(\left(n{\left(\frac{1}{2}\right)}^{log_{k}n-1}\right)\left(\log_{n}\left(w/n\right)\right)\right)$ with the exponentially high probability (=1−exp(−2/n)). Especially, if k=2, MaxRcvLoad is $O(log_{n}\left(w/n\right))$ with the exponentially high probability.

## 4. Experimental Results

In Section 4.1, we briefly describe the prototype implementation of DDR-coin using NetLogo, which is one of the most widely used agent-based modeling tools. In Section 4.2, we compare the analytic results of Section 3 and simulation results. In Section 4.3, we compare the previous work with DDR-coin using NetLogo. In Section 4.4, we discuss some issues of DDR-coin algorithm.

### 4.1. Prototype Implementation

In this section, we describe prototype implementation of the proposed algorithm, DDR-coin. We used NetLogo 6.1.1 (made by Northwestern University, IL, USA) [22] for the simulation. NetLogo is one of the most widely used agent-based simulation tools. It can be used for a wide range of topics, such as epidemic protocols, fractals, and topics in the social sciences [22]. In NetLogo, the Logo programming language is used for modeling. The source code for the prototype implementation is available at [23].

In NetLogo, simulations are conducted with discrete time steps called *ticks*. In the simulation of DDR-coin, a trigger is generated at each tick of the simulation. Each node is represented as an agent in the simulation. Each node (or agent) executes the algorithms in Section 2.4 at each tick. We assume that a message sent by a node arrives at the destination node instantaneously. It is also assumed that the order of messages sent from one node to another node during simulation is preserved. However, the order of messages sent from multiple nodes to different nodes may change. The message delay and loss will be handled in future work.

Our simulation code of DDR-coin has a main loop that runs repeatedly. In this main loop, a trigger is invoked and a node is randomly selected to get this trigger. After receiving this trigger, the node uses the algorithms in Section 2.4 to handle it: a coin message is generated with a predefined probability and then sent to another node as described in Section 2.4.

**Figure 6:**
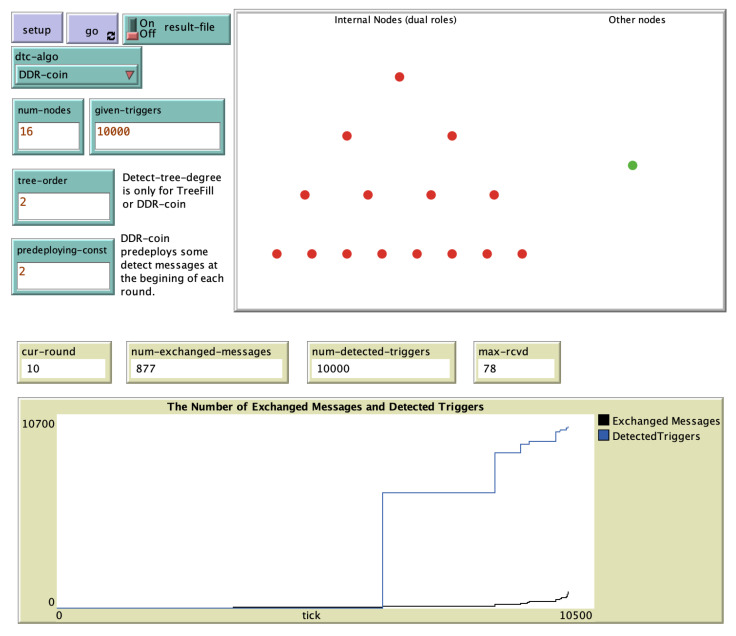
An example of DDR-coin simulation using NetLogo when $n=2^{4},\kappa =2$, and w=10,000.

In the set up procedure of the simulation, a *k*-ary tree-like structure is constructed. The number of nodes *n* is defined as $k^{L}$, where *k* and *L* can be selected by the user. In Figure 6, the simulation screenshot for $k=2,n=2^{4},\kappa =2,$ and w=10,000 is shown.

### 4.2. Comparison of Simulation Results with Mathematical Analysis

In the simulation of DDR-coin, we conducted experiments for various number of nodes while fixing κ and the number of triggers: w=10,000 and κ=5, which implies the failure probability is $4.472*10^{-5}\left(n=64\right)∼7.453*10^{-6}\left(n=2048\right)$. In our experiments, we used 3 values for *k* in *k*-ary tree-like structure: 2, 3, and 5. The number of nodes was $2^{i_{1}}$, $3^{i_{2}}$, and $5^{i_{3}}$, where $6\leq i_{1}\leq 11$, $4\leq i_{2}\leq 5$, and $3\leq i_{3}\leq 4$. We repeated 30 times to get the average value of message complexity, the number of rounds, and MaxRcvLoad.

**Figure 7:**
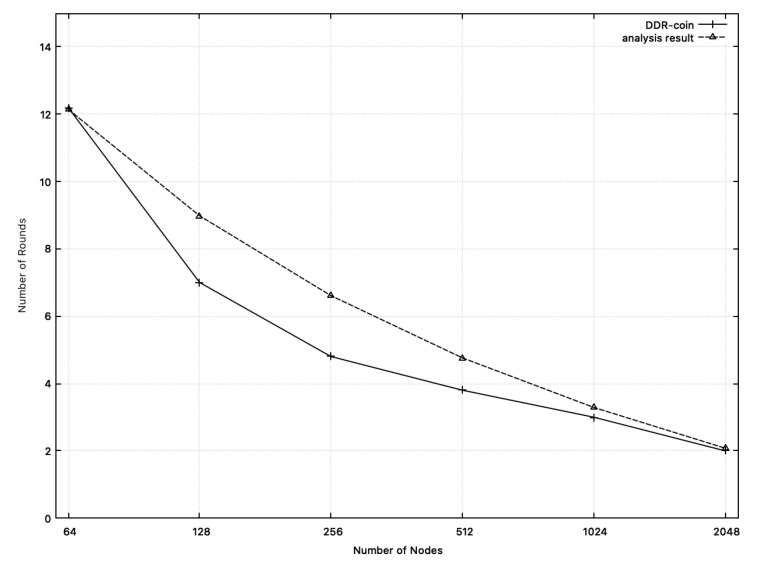
Numbers of rounds: the measured numbers from simulations and the analysis results from Section 3 when $n=2^{6}∼2^{11},w=10,000$. The solid line represents the measured numbers from simulation. The dotted line (analysis results) represents the function: $10\cdot \log_{n}\left(w/n\right)$.

**Figure 8:**
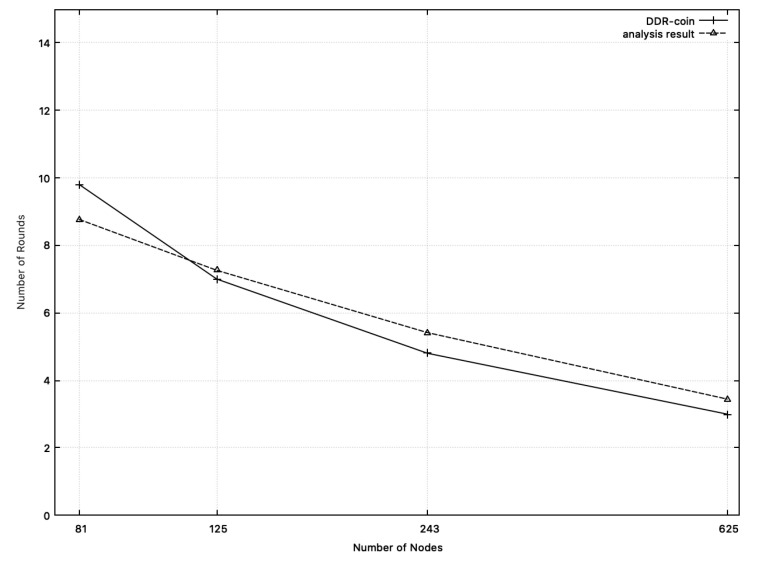
Numbers of rounds: the measured numbers from simulations and the analysis results from Section 3 when $n=3^{4},3^{5},5^{3},5^{4},w=10,000$. The solid line represents the measured numbers from simulation. The dotted line (analysis results) represents the function: $8\cdot \log_{n}\left(w/n\right)$.

**Number of rounds:**Figure 7 shows the comparison results between the measured number of rounds in simulation and the calculated one from analysis in Section 3. In this figure, we chose k=2,κ=5,w=10,000, and $n=2^{6}∼2^{11}.$ X-axis corresponds to the number of nodes while y-axis represents the number of rounds. In Figure 7, the dotted line represents the analysis results. Recall that the number of rounds analyzed in Section 3 is $O(\log_{n}\left(w/n\right))$. Among diverse functions for $O(\log_{n}\left(w/n\right))$, we choose $10\cdot \log_{n}\left(w/n\right)$ whose outputs are close to the measured numbers in simulation, which are represented in the solid line in Figure 7. Similarly, Figure 8 shows the comparison results on the number of rounds where k=3,w=10,000, κ=5 and $n=3^{4},3^{5},5^{3},5^{4}$. As shown in Figure 7 and Figure 8, the number of rounds from simulation results and that from analysis of Section 3 are close to each other.

**Figure 9:**
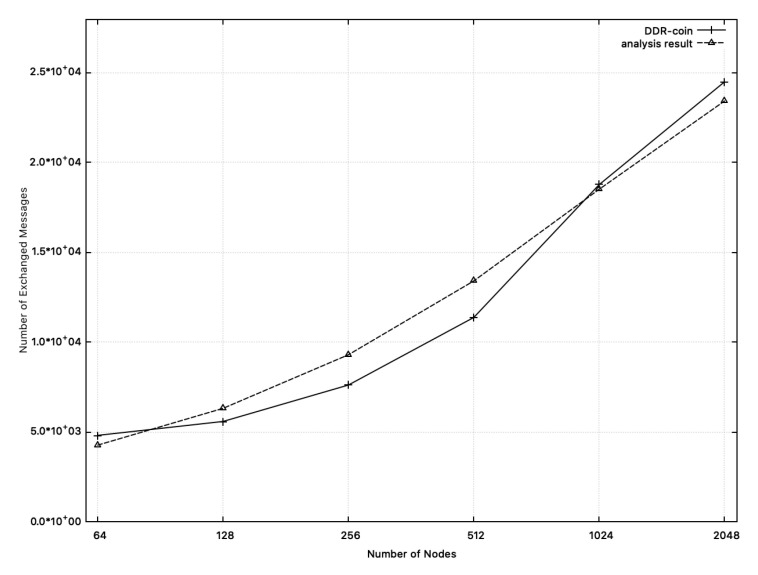
Message complexity: the measured numbers from simulations and the analysis results from Section 3 when $n=2^{6}∼2^{11},w=10,000$. The solid line represents message complexity measured from simulation. The dotted line (analysis results) represents the function: $55\cdot n\log_{n}\left(w/n\right)$.

**Figure 10:**
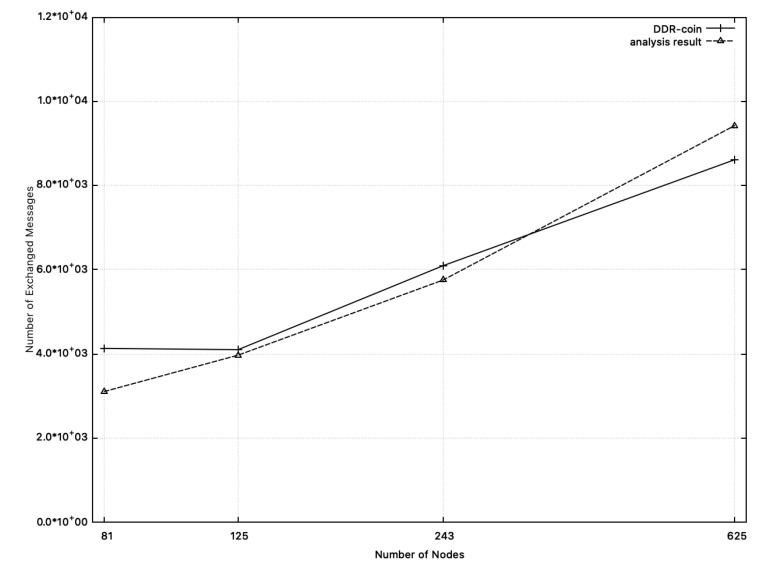
Message complexity: the measured numbers from simulations and the analysis results from Section 3 when k=3 or 5,w=10,000, and $n=3^{4},3^{5},5^{3},5^{4}$. The solid line represents message complexity measured from simulation. The dotted line (analysis results) represents the function: $35\cdot n\log_{n}\left(w/n\right)$.

**Message complexity:**Figure 9 shows the comparison results between the measured message complexity in simulation and the calculated one from analysis in Section 3. In this figure, we chose k=2,κ=5,w=10,000, and $n=2^{6}∼2^{11}.$ X-axis corresponds to the number of nodes where y-axis represents message complexity. In Figure 9, the dotted-line represents the analysis results. Recall that message complexity analyzed in Section 3 is $O(n\log_{n}\left(w/n\right))$. Among functions for $O(n\log_{n}\left(w/n\right))$, we choose $55\cdot n\log_{n}\left(w/n\right)$ whose outputs are close to the measured numbers in simulation, which are represented in the solid line in Figure 9. Similarly, Figure 10 shows the comparison results on message complexity where k=3 or 5,w=10,000, κ=5 and $n=3^{4},3^{5},5^{3},5^{4}$ (we chose $35\cdot n\log_{n}\left(w/n\right)$ for the dotted line). As shown in Figure 9 and Figure 10, message complexity from simulation results and that from analysis of Section 3 are close to each other.

**Figure 11:**
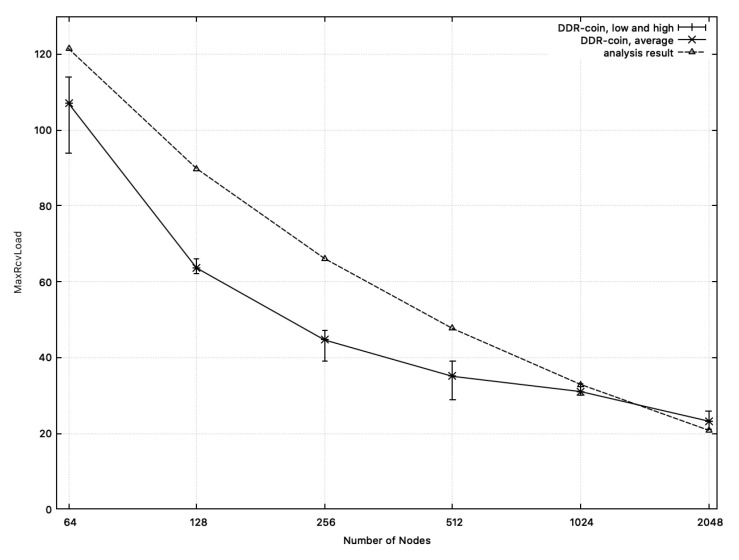
MaxRcvLoad: the measured numbers from simulations and the analysis results from Section 3 when $n=2^{6}∼2^{11},w=10,000$. The solid line represents MaxRcvLoad measured from simulation. The dotted line (analysis results) represents the function: $100\cdot \log_{n}\left(w/n\right)$.

**Figure 12:**
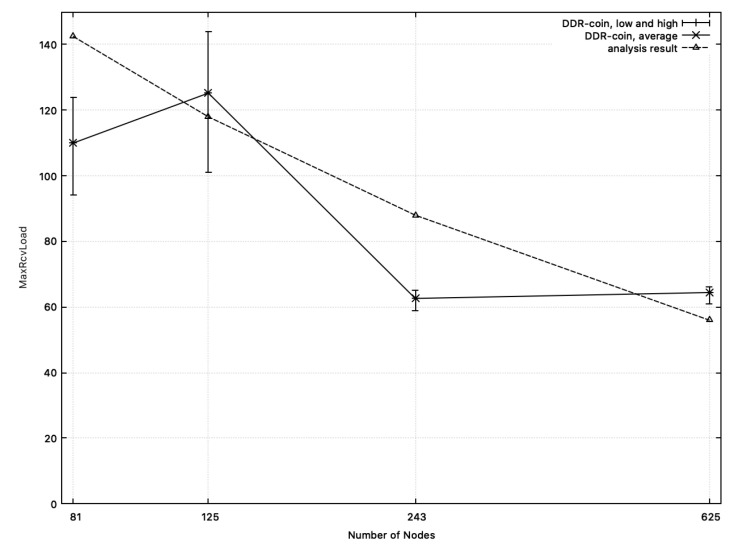
MaxRcvLoad: the measured numbers from simulations and the analysis results from Section 3 when k=3 or 5,w=10,000, and $n=3^{4},3^{5},5^{3},5^{4}$. The solid line represents MaxRcvLoad measured from simulation. The dotted line (analysis results) represents the function: $130\cdot \log_{n}\left(w/n\right)$.

**MaxRcvLoad:**Figure 11 shows the comparison results between the measured MaxRcvLoad from simulation and the calculated one from analysis in Section 3. In this figure, we chose k=2,κ=5,w=10,000, and $n=2^{6}∼2^{11}.$ X-axis corresponds to the number of nodes while y-axis represents MaxRcvLoad. In Figure 11, the dotted-line represents the analysis results. Recall that MaxRcvLoad analyzed in Section 3 is $O(\log_{n}\left(w/n\right))$. Among functions for $O(\log_{n}\left(w/n\right))$, we choose $100\cdot \log_{n}\left(w/n\right)$ whose outputs are close to the measured numbers in simulation, which are represented in the solid line in Figure 11. Similarly, Figure 12 shows the comparison results on the number of rounds where k=3 or 5,w=10,000, κ=5 and $n=3^{4},3^{5},5^{3},5^{4}.$ As shown in Figure 11 and Figure 12, MaxRcvLoad from simulation results and that from analysis of Section 3 are close to each other.

From measured message complexity, the average number of exchanged messages for each node is $35\cdot \log_{n}\left(w/n\right)∼55\cdot \log_{n}\left(w/n\right)$. Compared with this, the measured MaxRcvLoad, $100\cdot \log_{n}\left(w/n\right)∼130\cdot \log_{n}\left(w/n\right)$, is not so big, which implies that (roughly speaking) message load is evenly distributed among nodes.

### 4.3. Comparison with Previous Work

In this section, we compare the simulation results of DDR-coin with those of previous work. Among the previous schemes, we chose CoinRand [2], TreeFill [17], and RingRand [2], which show the best performance in terms of message complexity and MaxRcvLoad. In the simulation, we set the parameters as follows: w=10,000, $n=2^{i}$ (5≤i≤11), and κ=5, which implies the failure probability of DDR-coin is $1.178*10^{-4}$ (*n* = 32) $∼7.453*10^{-6}$ (*n* = 2048).

**Figure 13:**
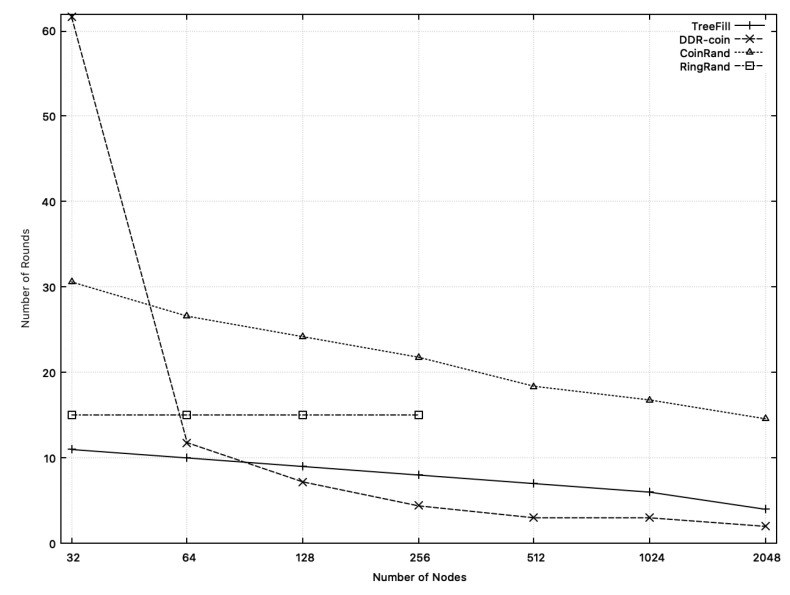
Comparison of the numbers of rounds of DDR-coin, TreeFill, CoinRand, and RingRand when the number of nodes are $2^{i}$ where 5≤i≤8. The number of triggers is w=10,000.

Figure 13 shows the number of rounds measured in simulations of TreeFill, DDR-coin, CoinRand, and RingRand. For n≤64, DDR-coin has the largest number of rounds since small *n* violates our assumption, $\kappa \ll \sqrt{n}$.

Except for this region, the number of rounds in DDR-coin is significantly smaller than that of CoinRand. CoinRand requires 2.3 to 7.3 times more than DDR-coin. We think that DDR-coin uses a complex tree-like structure and probabilistic algorithms, both of which reduce the number of rounds.

In Figure 13, if n≥64, the number of rounds in TreeFill is about 0.85 ∼ 2 times bigger than that of DDR-coin. If the number of nodes is relatively small (i.e., less than about 90), TreeFill has smaller number of rounds than DDR-coin. As the number of nodes increases, DDR-coin uses fewer rounds compared to TreeFill.

Note that in RingRand, the number of rounds is O(logw) for all *n* [2]. In the simulation results, the measured number of rounds is about 14∼15, which fits well with $\log_{2}\left(10,000\right)=13.3$. For n>256, we were unable to conduct experiments on RingRand due to rapid increase of message complexity.

**Figure 14:**
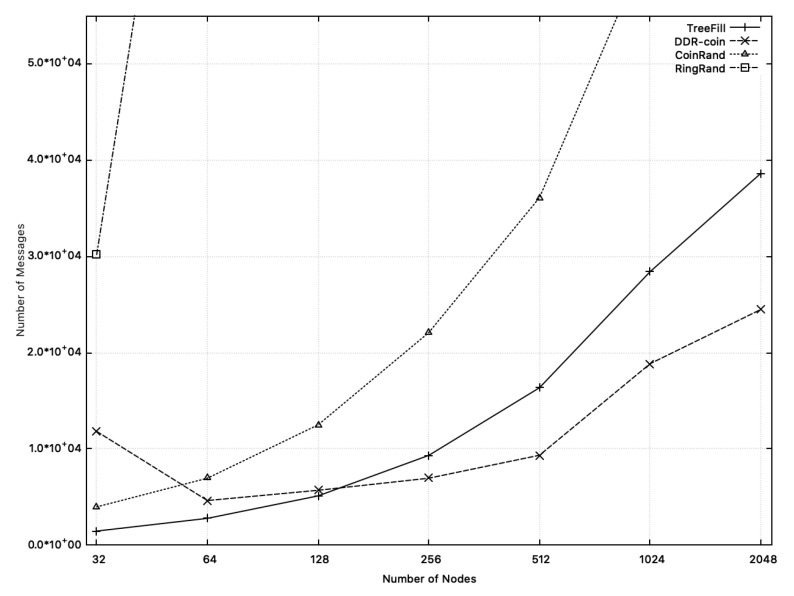
Comparison of the numbers of messages of DDR-coin, TreeFill, CoinRand, and RingRand when the number of nodes are $2^{i}$ where 5≤i≤10. The number of triggers is w=10,000.

Figure 14 shows the total number of messages used in TreeFill, DDR-coin, CoinRand, and RingRand. As shown in this figure, when n<152, among them TreeFill uses the smallest number of messages. For n≥152, DDR-coin has the smallest number of messages. As the number of nodes increases, the difference in message complexity also increases. Especially, RingRand shows the fastest increase.

In the case of CoinRand and DDR-coin, if n<64, DDR-coin uses more messages due to violation of our assumption, $\kappa \ll \sqrt{n}$. If the number of nodes increases, DDR-coin uses a much smaller number of messages than CoinRand. When the number of nodes is 256, DDR-coin uses about 1/3 less messages than CoinRand. If the number of nodes is 512, CoinRand uses about 4 times the messages compared to DDR-coin. The reason why CoinRand requires more messages is that CoinRand uses (about 2.3∼7.3 times) more rounds than DDR-coin.

TreeFill uses less messages than DDR-coin if n<152. For large *n*, DDR-coin uses fewer messages than TreeFill. When the number of nodes is 512, DDR-coin uses 68.4% of messages compared to TreeFill. From this, we think that TreeFill is better than DDR-coin for the case when the number of nodes is not so large.

**Figure 15:**
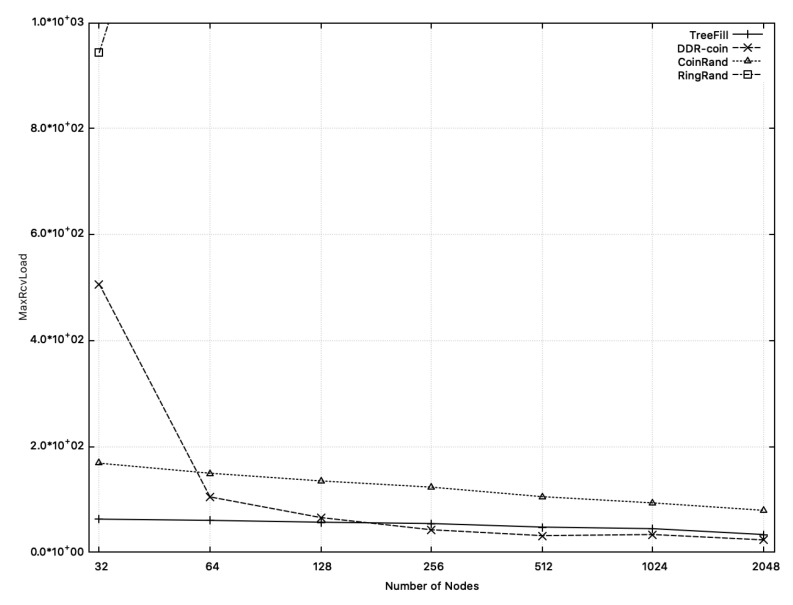
Comparison of MaxRcvLoad of DDR-coin, TreeFill, CoinRand, and RingRand when the number of nodes is $2^{i}$ where 5≤i≤8. The number of triggers is w=10,000.

Figure 15 shows the comparison of MaxRcvLoad of TreeFill, DDR-coin, CoinRand, and RingRand. For 64≤n≤2048, CoinRand uses 1.42 ∼ 3.37 times MaxRcvLoad compared to DDR-coin, and this difference increases as the number of nodes increases. MaxRcvLoad is affected by the number of rounds because it is the maximum of the number of messages received by each node while the algorithm is running. As shown in Figure 13, CoinRand requires 2.3 ∼ 7.3 times more the number of rounds than DDR-coin. DDR-coin uses more messages for each round than CoinRand but the number of rounds is smaller, which explains that MaxRcvLoad of DDR-coin is about 1.42 ∼ 3.37 times smaller compared to that of CoinRand.

TreeFill shows a smaller MaxRcvLoad than that of DDR-coin when *n* is less than about 180. However, as the number of nodes increases, DDR-coin uses fewer rounds than TreeFill, and thus MaxRcvLoad is also smaller than TreeFill. As for RingRand, MaxRcvLoad is much larger than other algorithms. We think that this is partially because our implementation is not fully optimized. Aside from implementation inefficiencies, we expect that MaxRcvLoad of RingRand is much higher than other algorithms since the analytic result of MaxRcvLoad is O(nlognlogw) [2], which is much higher than other schemes.

### 4.4. Discussion

In this subsection, we discuss some issues on DDR-coin algorithm: no-message drops, mean time to detect the global changes, relation of κ and message complexity, and demerit of DDR-coin. As for no-message drops, this assumption is adopted from most of the previous work [2,17,18] due to simplification of analysis. If the DTC algorithm is designed to allow message drops, message complexity will be higher and sometimes it has two-sided failures: even if less than *w* triggers are detected, it produces a false alarm. One of the easiest ways to allow some message drops is to establish reliable communication, e.g., challenge-and-response and retransmission. Otherwise, we can send coin messages for multiple nodes for redundancy, which also incurs extra communication overhead. (We leave the enhancement of DDR-coin to allow message drops while minimizing communication load for future work.)

For mean time to detect the global changes, inherently all DTC algorithms have some delay: when *w* triggers occurs, they detect this after some time. This is because all DTC algorithms have no false positives and focus on minimizing message complexity, MaxRcvLoad, and MaxMsgLoad. If we try to minimize this delay, it will cause additional communication overhead or lose accuracy. Therefore, this trade-off is another important research topic, which we also leave for future work.

**Figure 16:**
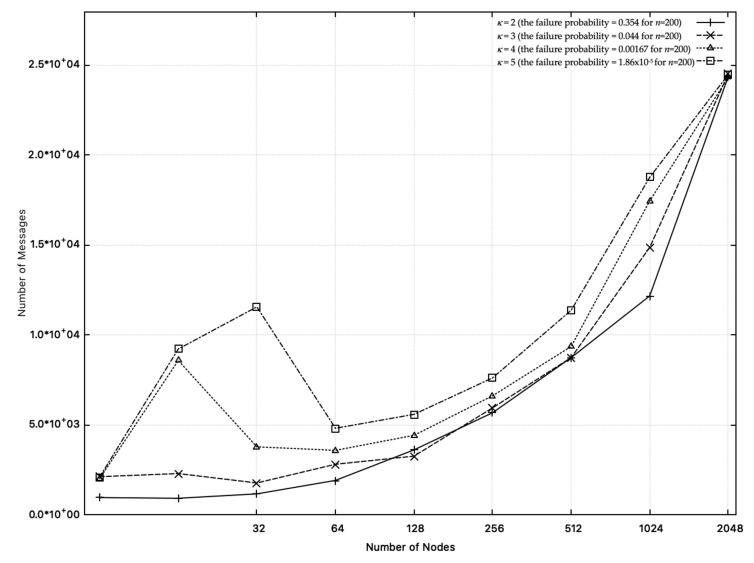
Message complexity over different κ values in DDR-coin: $n=2^{3}∼2^{11},w=10,000$.

κ affects the failure probability and message complexity. In DDR-coin algorithm, as κ is increased, message complexity also is increased, which is shown is Figure 16. (MaxRcvLoad has the similar property.) However, in the practical point of view, we do not need to use large κ: if the number of nodes is not too small and if we choose appropriate κ (e.g., κ = 4, 5, 6), the failure probability is extremely low while message complexity is much lower than the previously known best algorithms [2,17], which is shown in Section 4.3.

Compared to the previously-known best algorithms [2,17], the DDR-coin algorithm has the disadvantage that if *n* is not significantly greater than $\kappa^{2}$ (e.g., κ = 5, n≤ 32 ∼ 64), message complexity and MaxRcvLoad is similar or even bigger.

## 5. Related Work

DTC algorithms can be used as a building block for consistent global snapshots [3]. By using efficient DTC algorithms, the message complexity for storing global snapshots can be largely reduced compared with conventional global snapshot algorithms [12,13,14,15,16]. In conventional global snapshot algorithms, the message complexity of channel state recording is typically $O(n^{2})$. By using DTC algorithms, we can reduce the cost for channel state recording in global snapshots, where the message complexity is O(nlog(w/n)) [3].

Garg et al. proposed three DTC algorithms and proved the lower bound of message complexity for general DTC algorithms [3], where the lower bound of DTC algorithms is O(nlog(w/n)). One of their algorithms shows an optimal message complexity, but it uses a centralized approach and MaxRcvLoad of this DTC algorithm is not bounded.

Chakaravarthy et al. proposed a near optimal DTC algorithm called LayeredRand [1]. The message complexity and MaxRcvLoad of this algorithm are O(nlognlogw) and O(lognlogw), respectively [1]. In [2], they proposed two DTC algorithms, which can be considered as an improvement of [1]. The DTC algorithms they proposed are called CoinRand and RingRand, respectively [2]. The message complexity and MaxRcvLoad of CoinRand are O(n(logw+logn)) and O(logw+logn), respectively. This algorithm is based on a network topology similar with binary trees. They use a randomized technique in CoinRand during the message-aggregation process. As a result, it shows better performance than their previous work, LayeredRand [1]. The message complexity and MaxRcvLoad of RingRand are O(nlognlogw) and O(lognlogw), respectively.

Kim et al. proposed an optimal DTC algorithm [17]. The message complexity and MaxRcvLoad of their algorithm are O(nlog(w/n)) and O(log(w/n)), respectively. This is also based on a network topology similar with the tree structure.

Emek and Korman proposed DTC algorithms with more generalized assumptions on communications between nodes [18]. They proposed two DTC algorithms. The message complexity of one algorithm they proposed is $O(n\log w{\left(\log\log n\right)}^{2})$, but MaxRcvLoad of this algorithm is not analyzed. The message complexity and MaxRcvLoad of the other algorithm are $O(n{\left(\log w\log n\right)}^{2})$ and $O({\left(\log w\log n\right)}^{2})$, respectively.

Kshemkalyani proposed a hypercube-based algorithm for global snapshots [24]. The number of messages used in the hypercube-based algorithm is O(nlogn), which is lower than the optimal message complexity of DTC algorithms, O(nlog(w/n)). However, the message size in hypercube-based algorithm is O(n) whereas that of DTC algorithms is O(1).

Tsai proved the lower bounds of message complexity for global snapshot algorithms based on the general grid interconnection network, which is generalization of hypercube-based network [25].

Chang et al. proposed a DTC algorithm for arbitrary network topology [19]. The algorithm they proposed is mainly focused on wireless sensor networks (WSNs) in which network topology cannot be known in advance. In the worst case, their algorithm uses $x(n\left\lceil \log\frac{w-n}{n^{2}-n}/\log\frac{n}{n-1}\right\rceil +n^{2}-1)$ messages to solve the DTC problem, where *x* is twice the number of edges in a WSN.

## 6. Conclusions

In this paper, we proposed an efficient probabilistic Distributed Trigger Counting (DTC) algorithm, DDR-coin (Deterministic Detection of Randomly generated coins). Even though DDR-coin has a negligible (one-sided) failure probability, the number of exchanged messages to detect *w* trigger is lower than that of optimal deterministic DTC algorithms: the message complexity of DDR-coin is $O(n\log_{n}\left(w/n\right))$ on average and the MaxRcvLoad of DDR-coin is $O(\log_{n}\left(w/n\right))$ on average. We implemented prototype of DDR-coin using NetLogo 6.1.1 and then measured the message complexity and MaxRcvLoad to compare analytic results, which shows that the analytic results are close to the measured data. We also implemented CoinRand, RingRand, and TreeFill using NetLogo 6.1.1 for comparison. Experimental results show that DDR-coin shows the best performance for most of the cases. When the number of nodes is small, TreeFill is better than DDR-coin. In our experiments, message complexity and MaxRcvLoad of RingRand are greater than those of other algorithms. Our algorithm can be useful for taking global snapshots for large scale distributed systems and for detecting significant events in the distributed system with sensors. The future work includes precise analysis on the number of overflow-coin messages and implementation of library packages to cope with diverse real-life issues (including node failure, message delay/lost, and limitation on network topology).

## Figures and Tables

**Table 1:** Comparison of distributed trigger counting (DTC) algorithms.

Algorithm	Message	MaxRcvLoad	MaxMsgLoad	Exact or
	Complexity			Probabilistic
Centralized [3]	O(nlog(w/n))	−	−	Exact
Tree-based [3]	O(nlognlog(w/n))	O(lognlog(w/n))	O(lognlog(w/n))	Exact
LayeredRand [1]	O(nlognlogw)	O(lognlogw)	−	Exact
CompTreeRand [18]	$O(n\log w{\left(\log\log n\right)}^{2})$	−	−	Probabilistic
CompTreeDet [18]	$O(n{\left(\log w\log n\right)}^{2})$	$O({\left(\log w\log n\right)}^{2})$	$O({\left(\log w\log n\right)}^{2})$	Exact
CoinRand [2]	O(n(logw+logn))	O(logw+logn)	−	Exact
RingRand [2]	O(nlognlogw)	O(lognlogw)	O(lognlogw)	Probabilistic
TreeFill [17]	O(nlog(w/n))	O(log(w/n))	−	Exact
DDR-coin	$O(n\log_{n}\left(w/n\right))$	$O(\log_{n}\left(w/n\right))$	−	Probabilistic

(−: not bounded, which implies that the value is equal to the message complexity.) (The algorithms of the work in [18] are the bounds for arbitrary networks).

**Table 2:** Table of notation.

Category	Variable	Description
	*n*	The number of nodes.
Overall	*w*	The number of triggers to be detected.
	$\kappa (\ll \sqrt{n})$	Security parameter to adjust the failure probability (refer to Section 2.4.2 and Section 3.1).
	$w_{i}$	The number of remaining triggers to be detected at the beginning of Round *i*.
		$(w_{1}=w$, $w_{i}=w_{i-1}-\hat{w_{i-1}}\left(2\leq i\leq f\right))$.
Round *i*	$\hat{w_{i}}$	The number of detected triggers at Round *i*.
(1≤i≤f)	$w_{f}$	The number of remaining triggers in the beginning of the final (=*f*) round.
	*h*	The height of the tree-like structure.
	*k*	Each internal vertex has *k* children.
	$n_{j}$, node-*j*	Node *j* (1≤j≤n) corresponding to the vertex *j* in the tree-like structure.
	$n_{j}.trg$	The number of received triggers in $n_{j}$ (1≤j≤n).
	$d_{u}$	The node *u* for the internal vertex in the tree-like structure.
Tree-like	$d_{u}.cns\left[1..k\right]$	The Boolean array of length *k* in $d_{u}$.
structure	*coin*	When $n_{j}$ at level-*h* receives a trigger, it generates a *coin* message with the
		probability of $n/w_{i}$. This *coin* is sent to a randomly-selected node in level-(*h*-1).
	*full-coin*	When $d_{u}.cns\left[1\ldots k\right]$ becomes full (i.e., all entries are true),
		$d_{u}$ sends a *full-coin* to its parent.
		If a *coin* arrives at $d_{u}$ where $d_{u}.cns\left[1\ldots k\right]$ are already all true, $d_{u}$ sends
	*overflow-coin*	an *overflow-coin* to the randomly chosen node $d_{upper}$ at the upper level.
		(This coin will go up to a certain level and then go down to arrive
		at level-(*h*-1) eventually. Refer to Section 2.4.2)

## Appendix A

In this appendix we show a detailed example of Rounds 1 and 2 of our DDR-coin algorithm.

- Initial condition: w=81,n=9,κ=2. The tree-like structure is shown in Figure 1.
- Round 1 starts:
  – (Section 2.4.1:) $w_{1}=w=81$.
  – (Section 2.4.2:) $\kappa \sqrt{n}=2\sqrt{9}=6$ coins are pre-distributed at level-1, which is shown in Figure 3. As node-2 has 3 coins, its array, $d_{2}.cns\left[\right]$, is full and a *full-coin* is sent to the root (node-4).
  – (Section 2.4.1:) Note that in Round 1, for each detected trigger, a coin is generated with the prob. of $n/w_{1}=9/81=1/9$.
  – (Section 2.4.1:) Suppose that all nodes have detected 9 triggers, which implies that a coin is generated and sent to level-1, e.g., node-5.
  – (Section 2.4.1:) Suppose that all nodes have detected 9 triggers, which implies that a coin is generated and sent to level-1, e.g., node-8. Now, node-8’s array, $d_{8}.cns\left[\right]$, is full, so a *full-coin* is sent to node-4 (Section 2.4.2).
  – (Section 2.4.1:) Suppose that all nodes have detected 9 triggers, which implies that a coin is generated and sent to level-1, e.g., node-2. Since node-2 is already full, an *overflow-coin* is sent to node-4 (Section 2.4.2). This coin is forwarded to node-5. After receiving this coin, node-5’s array, $u_{5}.cns\left[\right]$, is now full. Hence, a *full-coin* is sent to the root, node-4.
  – The root, node-4, has received 3 *full-coins* and its array, $u_{4}.cns\left[\right]$, is full. (Section 2.4.2:) Therefore, node-4 initiates the end-of-round procedure.
  – In End-of-round procedure (Section 2.4.3), the root knows that in Round 1, $\hat{w_{1}}=27$ triggers have been detected. $w_{2}=w_{1}-\hat{w_{1}}=81-27=54$. It goes to Round 2.
- Round 2 starts:
  – (Section 2.4.1:) $w_{2}=54$.
  – (Section 2.4.2:) $\kappa \sqrt{n}=2\sqrt{9}=6$ coins are predistributed at level-1, which is shown in Figure 3. As node-2 has 3 coins, its array, $d_{2}.cns\left[\right]$, is full and a *full-coin* is sent to the root (node-4).
  – (Section 2.4.1:) Note that in Round 2, for each detected trigger, a coin is generated with the prob. of $n/w_{2}=9/54=1/6$.
  – (Section 2.4.1:) Suppose that all nodes have detected 6 triggers, which implies that a coin is generated and sent to level-1, e.g., node-5.
  – (Section 2.4.1:) Suppose that all nodes have detected 6 triggers, which implies that a coin is generated and sent to level-1, e.g., node-8. Now, node-8’s array, $d_{8}.cns\left[\right]$, is full, so a *full-coin* is sent to node-4 (Section 2.4.2).
  – (Section 2.4.1:) Suppose that all nodes have detected 6 triggers, which implies that a coin is generated and sent to level-1, e.g., node-2. As node-2 is already full, an *overflow-coin* is sent to node-4 (Section 2.4.2). This coin is forwarded to node-5. After receiving this coin, node-5’s array, $u_{5}.cns\left[\right]$, is now full. Therefore, a *full-coin* is sent to the root, node-4.
  – The root, node-4, has received 3 *full-coins* and its array, $u_{4}.cns\left[\right]$, is full. (Section 2.4.2:) Hence, node-4 initiates the end-of-round procedure.
  – In End-of-round procedure (Section 2.4.3), the root knows that in Round 1, $\hat{w_{2}}=18$ triggers have been detected. $w_{3}=w_{2}-\hat{w_{2}}=54-18=36$. It goes to Round 3.

## Appendix B

In this appendix, we show that when the number of nodes is *n*, the average number of overflow-coin messages sent in each round is O(n).

**Lemma** **A1.** 
*When a node receives a coin/an overflow coin from the lower level, the probability of sending an overflow-coin to the upper level is less than 1/2.*

**Proof.** **(sketch)** Recall that on the *k*-ary tree-like structure, the average number of coins arrives at the level-(*h*-1) nodes is *n*. First, we show that the probability of occurring an overflow-coin message when a coin arrives at a node at level-(*h*-1) is less than 1/2. There are n/k nodes in level-(*h*-1), and each node has an array of size *k*. When a coin arrives at a node, an overflow-coin occurs if the array is already full (i.e., all entries are true). The overflow-coin goes up to the upper level and eventually is put in an empty (=false) entry in the array of another node at level-(*h*-1). Because the node is randomly selected when going-up, this overflow-coin enters a randomly selected one of the empty entries in the arrays of level-(*h*-1) nodes. For the same *n*, if the *k* value in the *k*-ary tree-like structure decreases, the probability of generating an overflow-coin message increases (e.g., in the extreme case, if k=n, there is 1 node and the array size is *n* so no overflow occurs at all. If reduced to *k* = 3, the number of nodes is *n*/3. The array size of each node is 3. If 3 coins arrive at one node, it is full, and overflow occurs when another coin arrives. If *k* is further reduced to 2, the probability increases.). Consider the case where *k* = 1 (even though we cannot build the tree-like structure and this does not exactly match our scheme, we can still calculate the probability of occurring overflow because we only focus on the bottom level). If we show that the expected value of the probability of an overflow is less than 1/2, then, for all *k* > 1, we can see that this probability is also less than 1/2. Assume *k* = 1. There are *n* nodes at level-(*h*-1). Because k=1, the array size is 1 and when a coin arrives at a node, the array becomes full. When two or more coin messages are received for each node, overflow occurs and coins (except for the first one) are delivered to another nodes. When the first coin arrives at a node, the probability of overflow occurring is 0. When the second coin arrives, the probability that an overflow will occur is (1/n) because it accidentally goes to the node containing the first coin, and the overflow-coin goes to the randomly selected one of the not-yet fully filled nodes. When the third coin arrives, the probability that an overflow will occur is (2/n) (when entering one of the two nodes for the preceding two coins), and then the overflow-coin goes to the another not-yet fully filled node. Therefore, the expected value of the probability of occurring an overflow when *n* coins has arrived is (0∗1+1∗0)+((1/n)∗1+((n−1)/n)∗0)+((2/n)∗1+((n−2)/n)∗0)+…+((n−1)/n∗1+(1/n)∗0)=(1/2)(n−1)/n<1/2. Therefore, when k>1, the probability of overflow-coins occurring at level-(h-1) level is less than 1/2. Now, when an overflow-coin arrives at a node of level-(*h*-2), we show that the probability that the overflow coin will be forwarded to the upper level is also less than 1/2. There are $n/k^{2}$ nodes in level-(*h*-2), and the size of the array of each node is *k*. One entry in the array becomes true when a full-coin arrives. A full-coin means that the corresponding subtree is full (the array of all nodes of the subtree’s level-(*h*-1) is full with true). As mentioned in the above, as subtrees are randomly filled, a full-coin arrives randomly at one of the level-(*h*-2) nodes. (The total number of full-coins arriving is exactly n/k.) When an overflow-coin arrives at a node, the coin is forwarded to the upper level when the array is full (with true). For the same *n*, the probability of forwarding the overflow-coin increases as *k* becomes smaller, which is just the same as at level-(h-1), i.e., if the value of *k* increases, the sizes of each subtree grows and the probability that *k* subtrees are full decreases. For ease of analysis, we assume that *k* overflow-coins occur together and are processed together (actually, they are generated/processed one by one. When we analyze this for each overflow-coin we would complete full-proof, which we leave as future work). *k* overflow-coins arrive together at a randomly selected node of level-(*h*-2) and if it is full, it is eventually forwarded together to another node at level-(*h*-2) that is not yet full and then one entry in the array in that node is filled with the true value. Consider the case when *k* = 2. The number of nodes at level-(*h*-2) is n/4, the array size of each node is 2. The number overflow-coins arriving at level-(*h*-2) is at most n∗(1/2). Therefore, the number of 2-overflow-coins is n/4, so this is exactly the same case for the level-(*h*-1) when k=2 and the number of nodes is n/2. Thus, the probability of forwarding an overflow-coin is less than 1/2 for k=2 and when k>2, the probability that an overflow coin is forwarded is also less than 1/2. Similarly, it can be analyzed in the same way at all level-j(1≤j≤h−1) and the probability of forwarding an overflow-coin to the upper level is less than 1/2. □

From Lemma A1, we can prove the following theorem on the average number of overflow-coin messages.

**Theorem** **A1.** 
*When the number of nodes is n, the average number of overflow-coin messages in each round is O(n).*

**Proof.** Let $\bar{F}$ be the random variable which represents the number of overflow-coin messages when an overflow-coin is initially forwarded from a node in level-(h-1), repeatedly forwarded to a node in level-(h−m), and then eventually sent to a node in level-(h-1). From Lemma A1, when a coin is forwarded from level-(h-1) to level-(h−m) and to level-(h-1) again, the number of overflow-coin messages is 2m and the probability for this case is $Pr\left(\bar{F}=2m\right)<1/2^{m}$. We can get the bound of $E(\bar{F})$ as follows,

$$\begin{array}{c} E\left(\bar{F}\right)<\sum_{m=1}^{h}2m{\left(1/2\right)}^{m}=4-2^{1-h}h-2^{2-h}<4. \end{array}$$

Because nodes at level-(h-1) receive *n* coins (from level-*h*), $n\bar{F}$ is the number of coin forwardings when *n* coins are sent to nodes at level-(h-1), and $E(n\bar{F})<4n$. This implies that the average number of overflow-coins for each round is O(*n*). □
